# Uncovering subtype-specific metabolic signatures in breast cancer through multimodal integration, attention-based deep learning, and self-organizing maps

**DOI:** 10.1038/s41598-025-06459-y

**Published:** 2025-07-01

**Authors:** Parisa Shahnazari, Kaveh Kavousi, Hamid Reza Khorram Khorshid, Zarrin Minuchehr, Bahram Goliaei, Reza M. Salek

**Affiliations:** 1 Laboratory of Complex Biological Systems and Bioinformatics (CBB), Department of Bioinformatics, Institute of Biochemistry and Biophysics (IBB), University of Tehran, Tehran, Iran; 2 Genetics Research Center, University of Social Welfare and Rehabilitation Sciences, Tehran, Iran; 3 Personalized Medicine and Geno-Metabolics Research Center, Hope Generation Foundation, Tehran, Iran; 4 Department of Systems Biotechnology, National Institute of Genetic Engineering and Biotechnology, Tehran, Iran; 5 Institute of Biochemistry and Biophysics, Laboratory of Biophysics and Molecular Biology, Institute of Biochemistry and Biophysics (IBB), University of Tehran, University of Tehran, Tehran, Iran; 6 School of Clinical Medicine, University of Cambridge, Cambridge Biomedical Campus, Cambridge, CB2 0SP UK

**Keywords:** Multimodal Metabolomics, Breast Cancer Subtypes, Attention-Based Deep Learning, Self-Organizing Map, Metabolic Signatures, Feature Selection, SVM-RFE, XGB-RFE, Random Forest, Biochemistry, Cancer, Computational biology and bioinformatics, Systems biology, Biomarkers

## Abstract

This study integrates multimodal metabolomic data from three platforms—LC–MS, GC–MS, and NMR—to systematically identify biomarkers distinguishing breast cancer subtypes. A feedforward attention-based deep learning model effectively selected 99 significant metabolites, outperforming traditional static methods in classification performance and biomarker consistency. By combining data from diverse platforms, the approach captured a comprehensive metabolic profile while maintaining biological relevance. Self-organizing map analysis revealed distinct metabolic signatures for each subtype, highlighting critical pathways. Group 1 (ER/PR-positive, HER2-negative) exhibited elevated serine, tyrosine, and 2-aminoadipic acid levels, indicating enhanced amino acid metabolism supporting nucleotide synthesis and redox balance. Group 3 (triple-negative breast cancer) displayed increased TCA cycle intermediates, such as α-ketoglutarate and malate, reflecting a metabolic shift toward energy production and biosynthesis to sustain aggressive proliferation. In Group 4 (HER2-enriched), elevated phosphatidylcholines and phosphatidylethanolamines suggested upregulated mono-unsaturated phospholipid biosynthesis. The study provides a framework for leveraging multimodal data integration, attention-based feature selection, and self-organizing map analysis to identify biologically meaningful biomarkers.

## Introduction

Cancer is a complex, multifactorial disease arising from the intricate interplay of biological, molecular, environmental, and cellular factors. Current research focuses on early detection, biomarker discovery, and advanced therapeutic strategies to improve patient outcomes and extend survival^1,2^. Integrating diverse biological modalities has revolutionized cancer research, driving significant advancements in diagnostic accuracy, therapeutic innovation, and patient monitoring^3–5^. Notably, the combination of multimodal integration and machine learning has emerged as a transformative strategy, substantially enhancing the precision and accuracy of early cancer detection. Despite significant breakthroughs, numerous challenges persist in cancer research. Advanced computational techniques, such as Grammatical Evolution of Neural Networks and tensor fusion, hold substantial promise for integrating heterogeneous biomedical datasets. Nonetheless, their adoption is often limited by significant computational demands and the complexity associated with handling large-scale, high-dimensional data. In contrast, more accessible strategies, such as autoencoder-based deep learning and multi-kernel learning, have consistently yielded notable gains in classification performance across diverse biomedical domains^6,7^. However, these methods often depend on transforming the original feature space into compressed or latent representations to optimize model learning. While this transformation can boost predictive accuracy, it compromises interpretability by obscuring the biological significance of individual features. As a result, such approaches may fall short in identifying explicit biomarker profiles, which are critical for understanding cancer mechanisms, predicting clinical outcomes, and guiding therapeutic decisions. Developing integrative methodologies capable of managing high-dimensional data complexity while preserving feature interpretability and biological relevance remains essential. Achieving this balance is crucial to the reliable discovery of biomarkers and the advancement of precision medicine.

This study tackles these challenges by employing static and dynamic feature selection approaches both before and after multimodal integration to identify key metabolites that distinguish breast cancer subtypes. The objective was to isolate significant features capable of differentiating distinct subgroups using breast cancer tissue samples. A self-organizing map (SOM) was utilized to analyze metabolite contributions within each group, with the results validated through SOM-based heatmaps and statistical analyses.

A detailed overview of the machine learning methods and multi-class labeling definitions used in this study is provided below:

### Attention-based feature selection

Attention-based feature selection is an advanced methodology leveraging attention mechanisms within neural networks to dynamically identify and prioritize the most relevant input features for a given predictive or classification task^8–10^. In this study, by embedding attention mechanisms within a feed-forward neural network architecture, the approach assigns weights to input features, reflecting their relative importance in model predictions and dynamically selecting important features from heterogeneous datasets.

In a feed-forward neural network with attention, an attention mechanism operates as a differentiable layer that computes feature importance scores. These scores, often referred to as attention weights, indicate the contribution of each feature to the model’s output^11^. Formally, the attention mechanism can be expressed as follows:

1. **Input representation:** Let $X\in {R}^{n\times d}$ represent the input data, where n is the number of samples and d is the dimensionality of the feature space.
2. **Feature embedding:** Each feature xi in X is passed through an embedding layer or a transformation, producing a representation hi  ∈ Rk (where k is the embedding dimension).
  $${\mathbf{h}}_{{\mathbf{i}}} = {\text{ f }}\left( {{\mathbf{W}}_{{\text{h}}} {\text{x}}_{{\text{i}}} + {\mathbf{b}}_{{\text{h}}} } \right)$$
  where Wh and bh are the trainable weights and biases, and f (⋅) is a nonlinear activation function (e.g., ReLU).
3. **Attention scores:** Attention scores α_i_ are computed for each feature x_i_ by comparing its transformed representation hi against a learnable context vector u ∈ Rk. This is typically done using a dot product followed by a softmax normalization:
  $${\upalpha }_{{\text{i}}} = { }\frac{{\exp \left( {{\mathbf{u}}^{T} {\varvec{h}}_{i} } \right)}}{{\mathop \sum \nolimits_{j = 1}^{d} \exp \left( {{\varvec{u}}^{T} {\varvec{h}}_{j} } \right)}}$$
  Here, αi∈ [0,1] represents the attention weight for the i-th feature, and the sum of all attention weights is constrained to 1 for interpretability.
4. **Weighted feature aggregation:** The attention weights are used to compute a weighted sum of the feature representations, producing an output vector z:
  $$z = \mathop \sum \limits_{i = 1}^{d} \alpha_{i} h_{i}$$
  This aggregated representation **z** serves as the input to subsequent layers of the neural network for prediction tasks.

In contrast to conventional static feature selection methods, which rely on predefined criteria to select features before model training, dynamic feature selection adapts during the training process. This adaptability enables attention-based models to adjust their focus based on the data’s characteristics, allowing for a more nuanced understanding of the underlying biological processes^12^.

### Self-organizing map (SOM)

SOM is a type of artificial neural network designed for unsupervised learning, often used for clustering and visualizing high-dimensional data. SOM consists of an input layer connected to a two-dimensional grid of neurons in the output layer, where each neuron is associated with a weight vector representing the input space^13^. During training, for each input vector, the SOM identifies the Best Matching Unit (BMU)—the neuron whose weight vector is closest to the input vector, usually based on Euclidean distance. Once the BMU is identified, its weights and those of neighboring neurons are updated to more closely resemble the input vector. This adjustment follows the rule:

$${\text{New Weight }} = {\text{ Old Weight }} + \, \alpha \left( {\text{t}} \right) \, *{\text{ h}}\left( {{\text{c}},{\text{ t}}} \right) \, * \, \left( {{\text{X }} - {\text{ Old Weight}}} \right)$$

$${\text{New Weight }} = {\text{ Old Weight }} + \, \alpha \left( {\text{t}} \right) \, *{\text{ h}}\left( {{\text{c}},{\text{ t}}} \right) \, * \, \left( {{\text{X}} - {\text{Old Weight}}} \right)$$

Where:

- ***α(t)*** is the learning rate, which typically decreases over time.
- ***h (c, t)*** is the neighborhood function for node ***c*** at time ***t***.
- ***X*** is the input vector.

This process, repeated over many iterations, enables the SOM to map similar input patterns to nearby neurons, preserving the topological relationships in the data and clustering similar patterns together.

In a supervised SOM, class labels are incorporated into the training, where the model learns to associate regions of the output map with specific classes. While still organizing based on input features, the model refines its ability to recognize boundaries between different classes, thus combining clustering and classification in a single, interpretable framework^14–16^.

### Breast cancer subtype classification (Based on Hormone Receptor and HER2 Status):

In the absence of the Ki67 biomarker in most breast cancer tissue samples in this study, the conventional molecular classification of subtypes could not be applied^17,18^. Consequently, the breast cancer subtypes were redefined into four distinct groups:

- Group 1: Estrogen Receptor (ER) or Progesterone Receptor (PR) positive, and Human Epidermal Growth Factor Receptor 2 (HER2) negative.
- Group 2: ER, PR, and HER2 positive (Triple positive).
- Group 3: ER, PR, and HER2 negative (Triple negative).
- Group 4: ER and PR negative, but HER2 positive (HER2-enriched).

## Results

### Workflow

**Fig. 1:**
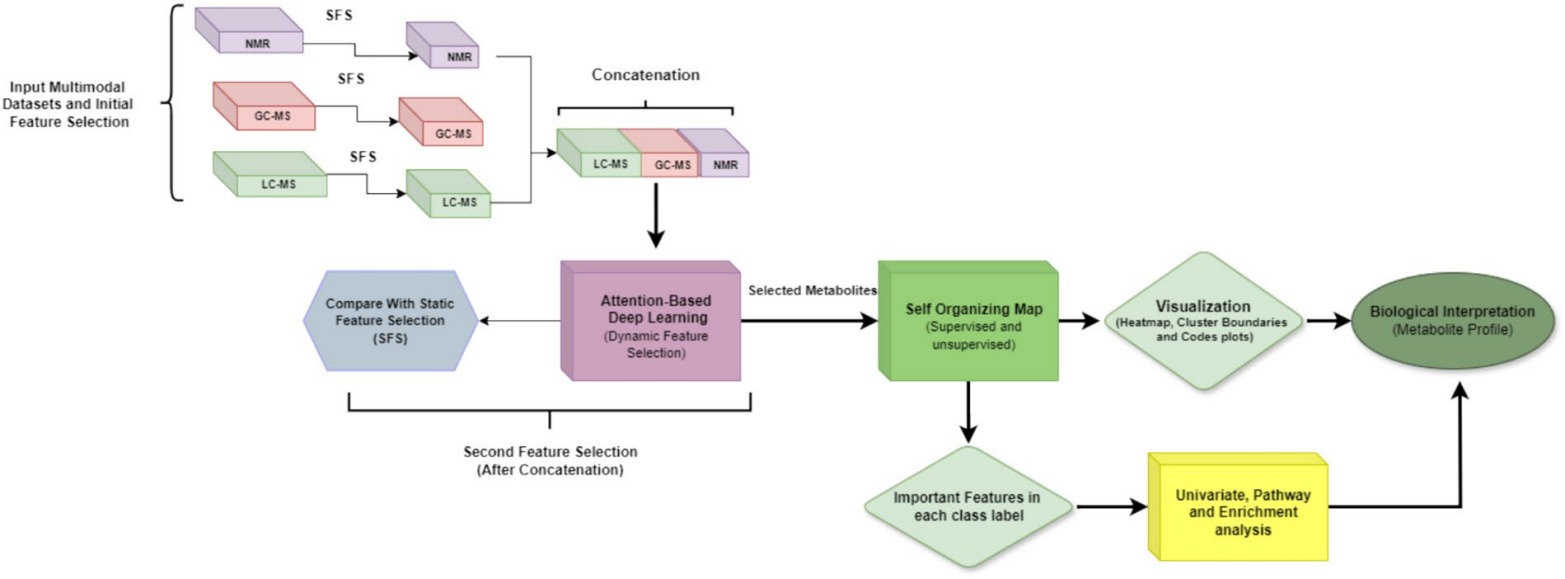
Workflow diagram for identifying metabolite signatures in breast cancer subtypes. The SVM-RFE feature selection is initially applied to each metabolomics platform to identify significant features. Following data concatenation and normalization, attention-based feature selection is performed to identify significant metabolites. SOM analysis is then utilized to visualize the contributions of each class label, complemented by SOM heatmaps annotated with statistical significance. The identified features are biologically interpreted as metabolite signatures, providing insights into distinct breast cancer subtypes.

The overall workflow is presented in Figure 1. Multimodal metabolomics datasets from three platforms (LC-MS, GC-MS, and NMR) were curated, preprocessed, and subjected to oversampling to address class imbalance. Feature selection was conducted in two phases. Support Vector Machine-Recursive Feature Elimination (SVM-RFE) was applied to each modality in the first phase. In the second phase, the selected features from each modality were concatenated into a unified dataset, with each sample associated with corresponding multiclass labels (four defined groups). Dynamic feature selection was then performed using a custom Feed-Forward Attention-based Deep Learning (F-ADL) model, which resulted in the selection of 99 metabolites. These were compared to the results from static feature selection methods, including SVM-RFE, Extreme Gradient Boosting Recursive Feature Elimination (XGBoost-RFE), and Random Forest (RF), which selected 100 metabolites. Further analysis was conducted using SOM and other visualization techniques to explore the relationships between the selected features and the class labels. Finally, the identified metabolites were analyzed through univariate analysis, pathway analysis, SOM heatmaps, and enrichment analysis to interpret their biological significance. This comprehensive approach led to the identification of biomarker profiles for each group.

### Preprocessing, oversampling, and initial feature selection

Each platform-specific dataset (LC-MS, GC-MS, and NMR) was preprocessed independently: missing values were imputed, features were min–max scaled to [0,1], and samples were row-wise L2-normalized to unit length to ensure comparability. To address class imbalance across the four clinical subgroups while preserving biological consistency, SMOTE was applied to each dataset, expanding each from 253 to 740 samples (185 per subgroup). Next, initial feature selection was performed using SVM-RFE (C = 1), yielding 92 LC-MS, 94 GC-MS, and 95 NMR features. Finally, these feature sets were concatenated into a unified 740-sample dataset.

### Concatenation of multimodal feature-selected datasets

Selected features from the three metabolomics platforms were horizontally concatenated into a unified dataset, with each sample characterized by a multiclass group label and features extracted from the three heterogeneous data sources.

### Attention-based feature selection

**Fig. 2:**
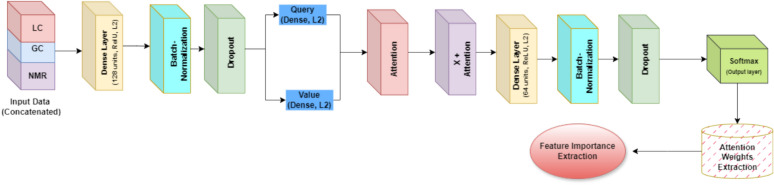
The Attention-based Feature Selection workflow. A custom Feed-Forward Attention-based Deep Learning (F-ADL) is applied for multiclass classification, incorporating an attention mechanism with separate query and value layers to highlight the most relevant features. The model undergoes hyperparameter tuning via a randomized search, optimizing dropout rates, L2 regularization, epochs, and batch sizes. Performance is evaluated using a variety of metrics, ensuring robust model assessment.

The architecture of the F-ADL model, summarized in Figure 2. began with data concatenation. The dataset was then divided into training and test sets. A custom Feed-Forward Neural Network with Attention was defined, consisting of a dense layer with 128 units, followed by dropout layers with rates of 0.6, 0.7, and 0.8. These rates were optimized through random search hyperparameter tuning, with higher dropout rates applied specifically to mitigate overfitting. In the attention-based segment of the model, dynamic feature selection was performed using query and value mechanisms, effectively identifying the most relevant features within the data. The model achieved optimal performance by combining the output of the attention mechanism with the original concatenated features, emphasizing key features through the learned attention weights. A subsequent dense layer with 64 units, incorporating L2 regularization, was applied, followed by additional dropout layers. For classification, the final layer employed a softmax function. Feature importance was derived from the attention weights, enabling the identification of critical features within the F-ADL model.

**Fig. 3:**
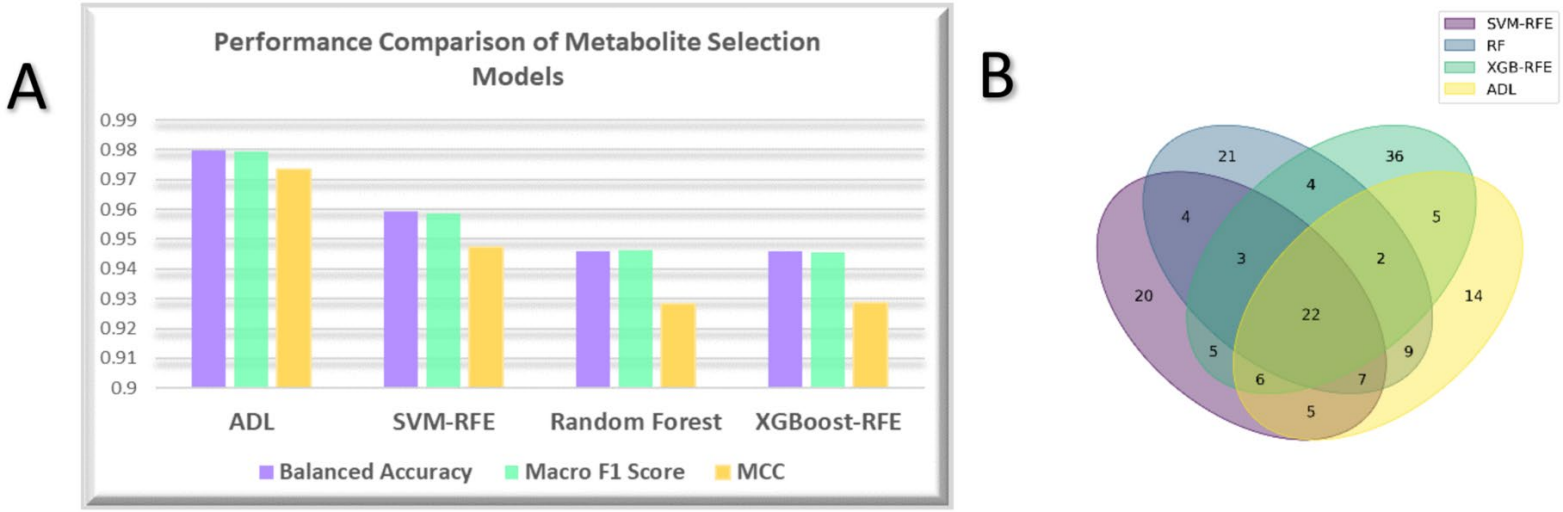
Comparison of feature elimination approaches. (**A**) Comparison of Balanced Accuracy, Macro F1 Score, and MCC between dynamic and static feature selection models. (**B**) Venn diagram illustrating the unique and shared metabolites identified by each feature selection model.

Table 1 indicates the evaluation metrics for the F-ADL model compared to three static feature selection methods—SVM-RFE, RF, and XGBoost-RFE—across cross-validation, training, and test sets. The F-ADL model consistently outperformed all static methods across all evaluation metrics. It achieved near-perfect performance on the training set and demonstrated high predictive reliability on the test set, with both accuracy and balanced accuracy reaching 0.9865. The model’s robustness was further reflected by a low Hamming loss of 0.0135 and a high Matthews correlation coefficient (MCC) of 0.9822.SVM-RFE ranked second, achieving accuracy and balanced accuracy of 0.9595 on the test set, an MCC of 94.75%, and a Hamming loss of 4.05%, demonstrating strong classification performance. In contrast, the overall performance of RF and XGB-RFE was suboptimal across all evaluation metrics on the test set. Despite efforts to enhance performance and reduce overfitting by adjusting parameters and increasing L2 regularization, the consistency of results across the training, test, and cross-validation sets remained inferior. Figure 3A compares the balanced accuracy, macro-F1 score, and MCC of the F-ADL model against the static models, highlighting the F-ADL performance advantage. Figure 3B presents a Venn diagram illustrating the intersections and unique metabolites identified by each model, with redundant metabolites excluded for clarity. The F-ADL model identified the largest number of common metabolites (56), followed by SVM-RFE with 52. However, F-ADL identified fewer unique metabolites than the other models, while XGBoost-RFE had the highest count of unique metabolites.

**Table 1:** Performance Comparison of the ADL Model and Static Feature Selection Methods (SVM-RFE, Random Forest, XGBoost-RFE) across Cross-validation, Training, and Test Sets.

Metrics	Cross Validation	Train	Test
F-ADL			
Accuracy	0.9975 ± 0.0054	0.9983	0.9865
Balanced Accuracy	0.9974 ± 0.0053	0.9983	0.9865
Macro F1 Score	0.9975 ± 0.0054	0.9983	0.9864
Micro F1 Score	0.9975 ± 0.0054	0.9983	0.9865
Weighted F1 Score	0.9975 ± 0.0054	0.9983	0.9864
Hamming Loss	0.0025 ± 0.0054	0.0017	0.0135
MCC	0.9966 ± 0.0051	0.9977	0.9822
SVM-RFE			
Accuracy	0.9425 ± 0.0247	0.9595	0.9595
Balanced Accuracy	0.9423 ± 0.0248	0.9595	0.9595
Macro F1 Score	0.9400 ± 0.0260	0.9588	0.9585
Micro F1 Score	0.9425 ± 0.0247	0.9595	0.9595
Weighted F1 Score	0.9399 ± 0.0260	0.9588	0.9585
Hamming Loss	0.0575 ± 0.0247	0.0405	0.0405
MCC	0.9261 ± 0.0317	0.9464	0.9475
Random Forest			
Accuracy	0.9662 ± 0.0191	0.9903	0.9459
Balanced Accuracy	0.9662 ± 0.0191	0.9903	0.9459
Macro F1 Score	0.9662 ± 0.0193	0.9904	0.9463
Micro F1 Score	0.9662 ± 0.0191	0.9903	0.9459
Weighted F1 Score	0.9662 ± 0.0193	0.9904	0.9461
Hamming Loss	0.0338 ± 0.0000	0.0097	0.0541
MCC	0.9553 ± 0.0253	0.9872	0.9284
XGBoost-RFE			
Accuracy	0.9105 ± 0.0045	0.9780	0.9459
Balanced Accuracy	0.9103 ± 0.0047	0.9780	0.9459
Macro F1 Score	0.9093 ± 0.0047	0.9780	0.9455
Micro F1 Score	0.9105 ± 0.0045	0.9780	0.9459
Weighted F1 Score	0.9093 ± 0.0048	0.9780	0.9455
Hamming Loss	0.0895 ± 0.0045	0.0220	0.0541
MCC	0.8819 ± 0.0058	0.9707	0.9286

The F-ADL model consistently outperforms the static methods in all evaluation metrics, including accuracy, balanced accuracy, MCC, and Hamming loss, highlighting its superior generalization and robustness.

### Self-Organizing map (SOM) and metabolite signatures

**Fig. 4:**
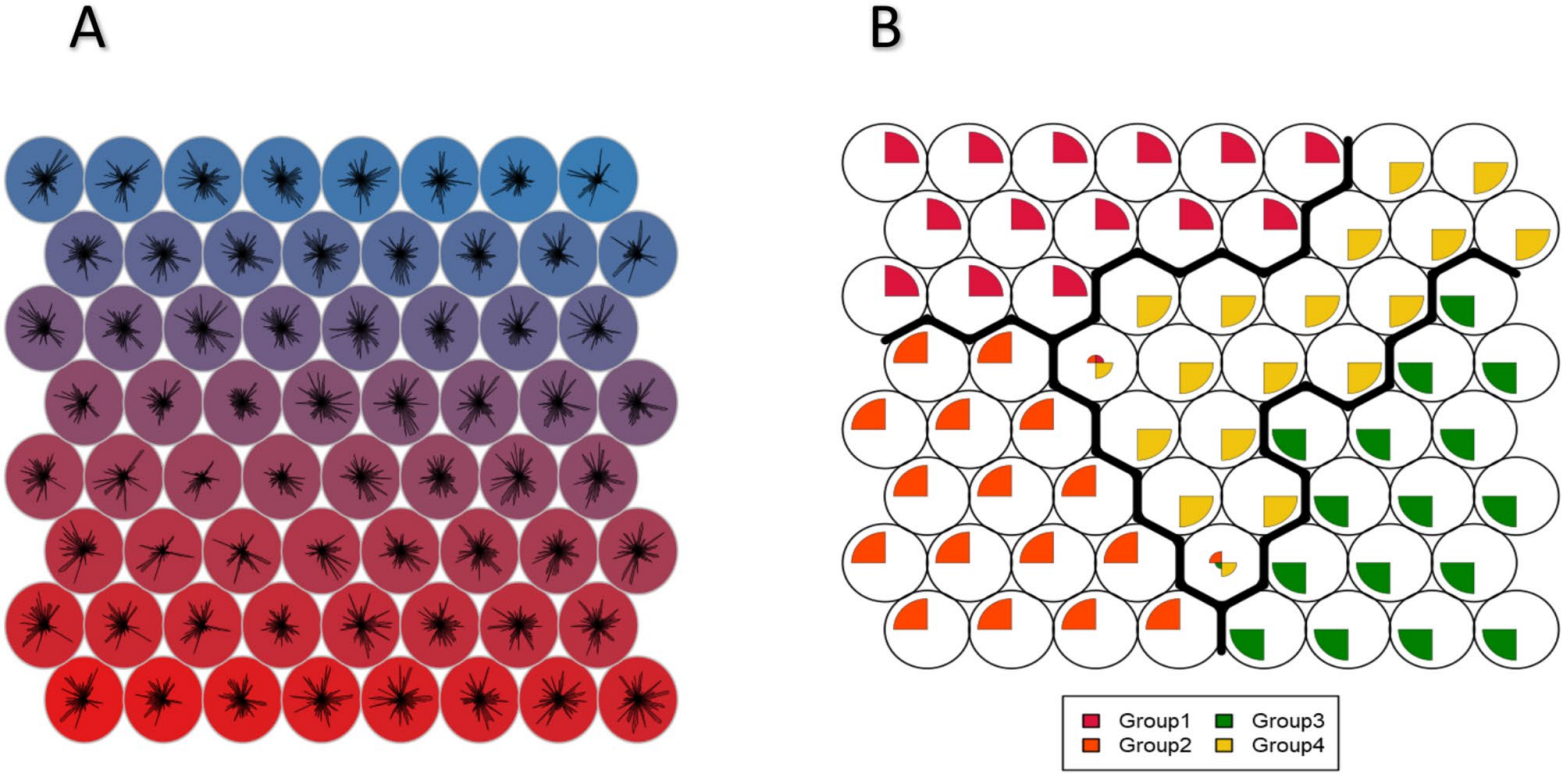
Visualization of Unsupervised and Supervised SOM Models. (**A**) Metabolite contributions across Self-Organizing Map (SOM) nodes are depicted using star plots. Each circle represents a SOM node, with rays corresponding to individual selected metabolites. The length of each ray indicates the relative magnitude of that metabolite’s contribution at the respective node. The number of rays is consistent across nodes and reflects the total number of selected metabolites included in the analysis(similar patterns cluster). Background colors, derived from a perceptually distinct palette, are used solely to enhance visual differentiation across the map and do not represent predefined clusters or class labels. (**B**) Class boundaries from the supervised SOM model trained with known group labels. Each node is assigned to a class based on the most frequent class among its mapped samples. Boundary lines indicate transitions between different class regions. The triple-negative group (Group 3) shows enhanced separation with minimal overlap, highlighting distinct feature representation.

The selected metabolites from the concatenated data, derived using the Attention model, were employed as input for the Self-Organizing. Figure 4A highlights the distribution of these metabolites across four clusters on the 2D map. Consistency in feature contributions was observed, except for a few nodes with sparse metabolite representation. In Figure 4B, the four multiclass groups—Group1, Group2, Group3, and Group4—are delineated by distinct boundaries. The class labels are accurately mapped to the SOM nodes. However, some overlap is seen between Group2 (ER, PR, and HER2 positive) and Group4 (HER2 rich) in certain nodes, which is biologically plausible due to shared HER2 reprogramming pathways. Additionally, minor overlaps of Group4 with Group3 and Group1 are noted, though these are less prominent and harder to detect.

**Fig. 5:**
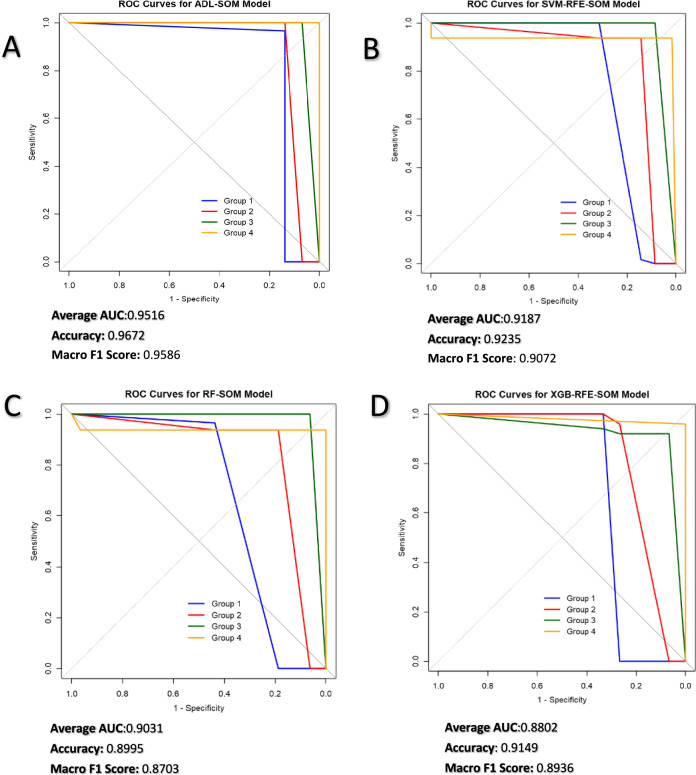
Evaluation of SOM classification performance across feature‐selection methods. The classification performance of various feature‐selection strategies was evaluated using a Self‐Organizing Map (SOM) classifier for breast cancer subtype prediction. Panels A–D illustrate receiver‐operating‐characteristic (ROC) curves for four feature‐selection approaches: (**A**) ADL-SOM (attention-driven feature selection), (**B**) SVM-RFE-SOM (support-vector-machine recursive feature elimination), (**C**) RF-SOM (random-forest-based feature selection) and (**D**) XGB-RFE-SOM (XGBoost-based recursive feature elimination). Among all methods, ADL-SOM achieved the highest performance, while SVM-RFE-SOM led among the static approaches. Evaluation metrics include average area under the curve (AUC), accuracy and macro F₁-score. ROC curves represent SOM classification results for the four breast cancer subtypes (Groups 1–4), with line colours corresponding to subtype labels as indicated in the legend.

The classification performance of the supervised SOM, defined by boundaries, was evaluated using the average AUC, accuracy, and Macro F1 score derived from data selected by the F-ADL method and traditional static methods **(**Figure 5**)**. The F-ADL approach outperformed all other methods, achieving an accuracy of 0.9672, an AUC of 0.9518, and a Macro F1 score of 0.9586.

**Fig. 6:**
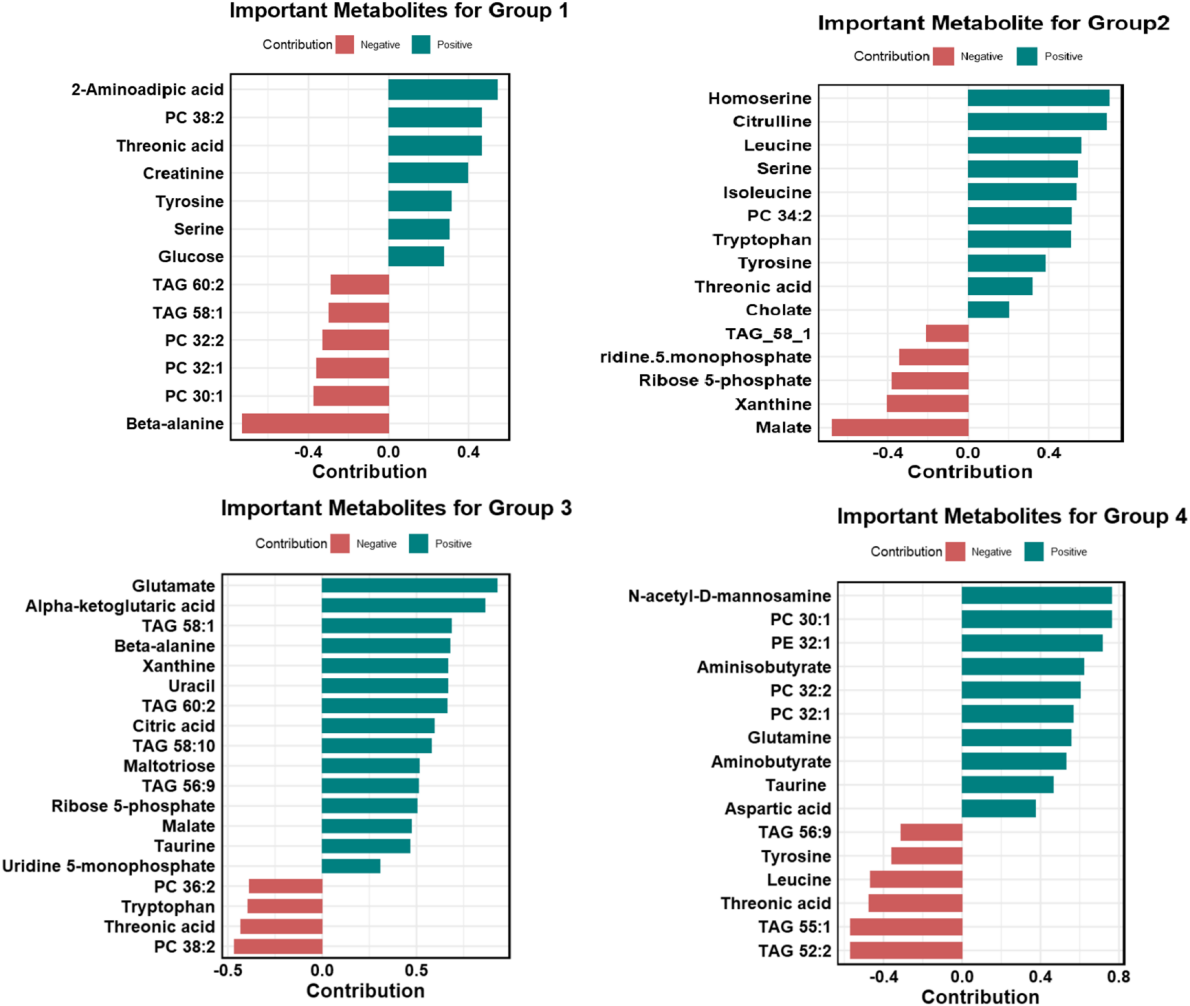
Group-specific contributions of key metabolites identified through Self-Organizing Map analysis. Each bar illustrates the average component weight (codebook vector value) of a metabolite across SOM nodes that are predominantly associated with a specific group. Positive values (dark cyan) indicate elevated metabolite levels, while negative values (Indian red) signify reduced levels in the corresponding group. These contributions reflect the strength of the association between each metabolite and the metabolic patterns characteristic of each group.

Figure 6 presents the metabolite contributions within the SOM model, applied attention-based feature selection, for each multiclass label group. An accompanying enrichment plot highlights significant variation across the four defined groups. In Group 1 (ER or PR positive and HER2 negative), metabolites with positive contributions include 2-Aminoadipic acid, PC 38:2, Threonic acid (0.464), Creatinine (0.396), Tyrosine (0.312), and Serine (0.304). Conversely, metabolites with negative contributions include TAG 60:2 (-0.286), TAG 58:1 (-0.296), PC 32:1 (-0.359), PC 32:2 (-0.328), PC 30:1 (-0.372), and Beta-alanine (-0.728).In Group 2, the key metabolites with positive contributions include Tyrosine (0.383), Isoleucine (0.536), Leucine (0.561), Cholate (0.721), Tryptophan (0.520), Threonic acid (0.510), Homoserine (0.700), Citrulline (0.684), PC 34:2 (0.512), and Serine (0.542). Negative contributors include Ribose 5-phosphate (-0.382), Xanthine (-0.401), TAG 58:1 (-0.208), Uridine-5-monophosphate (-0.343), and Malate (-0.678). Common positive contributors between Group 1 and Group 2 include Threonic acid, Tyrosine, and Serine, while TAG 58:1 is a shared negative contributor. In Group 3 (triple-negative breast cancer), the contribution profile demonstrates distinct differences compared to the other groups. Positive contributors include TAG 58:1 (0.686), TAG 60:2 (0.664), TAG 58:10 (0.578), TAG 56:9 (0.510), Xanthine (0.667), Uridine 5-monophosphate (0.308), Glutamate (0.926), Citric acid (0.596), Alpha-ketoglutaric acid (0.864), Maltotriose (0.514), Beta-alanine (0.6773), Ribose 5-phosphate (0.5036), Uracil (0.667), Malate (0.473), and Taurine (0.464). Negative contributors include Threonic acid (-0.433), Tryptophan (-0.394), PC 36:2 (-0.385), and PC 38:2 (-0.465).

Notably, Beta-alanine, Alpha-ketoglutaric acid, and Glutamate exhibited a significant increase in positive contribution, underscoring their critical role in Group 3’s metabolic landscape. The pronounced contributions of specific triacylglycerols (TAG), including TAG 58:1, TAG 60:2, and TAG 58:10, further distinguish Group 3 from the other groups, highlighting its unique metabolic profile within the triple-negative breast cancer subtype.

In Group 4 (HER2-rich), the most significant positive contributors include PC 30:1 (0.762), PC 32:1 (0.567), PC 32:2 (0.603), PE 32:1 (0.715), N-acetyl-D-mannosamine (0.763), aspartic acid (0.373), aminobutyrate (0.529), aminoisobutyrate (0.622), taurine (0.464), and glutamine (0.5543). Negative contributors include TAG 55:1 (-0.570), TAG 52:2 (-0.570), threonic acid (-0.475), tyrosine (-0.358), leucine (-0.469), and TAG 56:9 (-0.3114). Notably, N-acetyl-D-mannosamine (0.763) and PC 30:1 (0.762) are uniquely significant to Group 4, exhibiting the highest positive contributions compared to other groups. Additionally, metabolites such as PC 32:1, PC 32:2, and PE 32:1 show strong contributions in Group 4 but are absent in the other groups, underscoring their distinct role in the HER2-rich subtype.

**Fig. 7:**
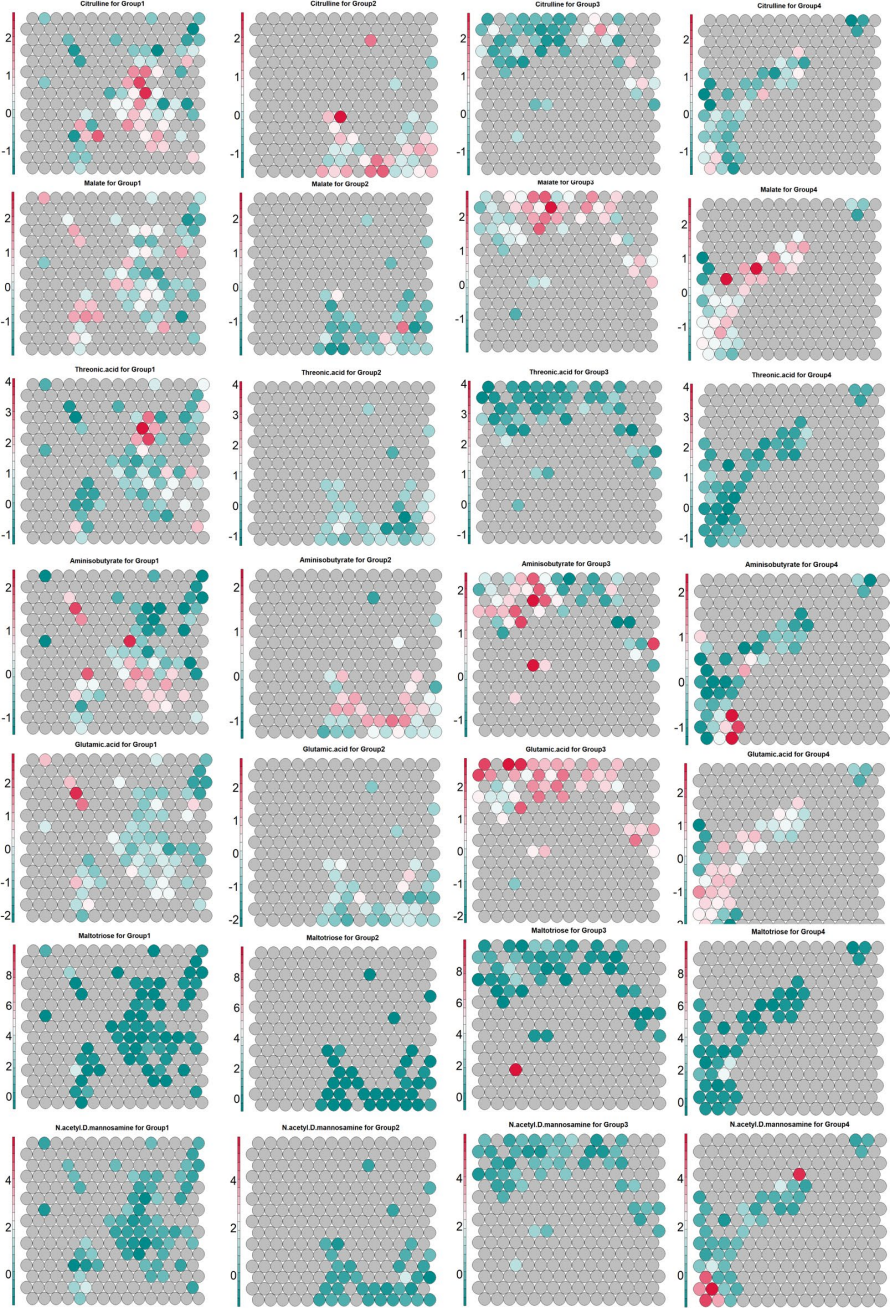

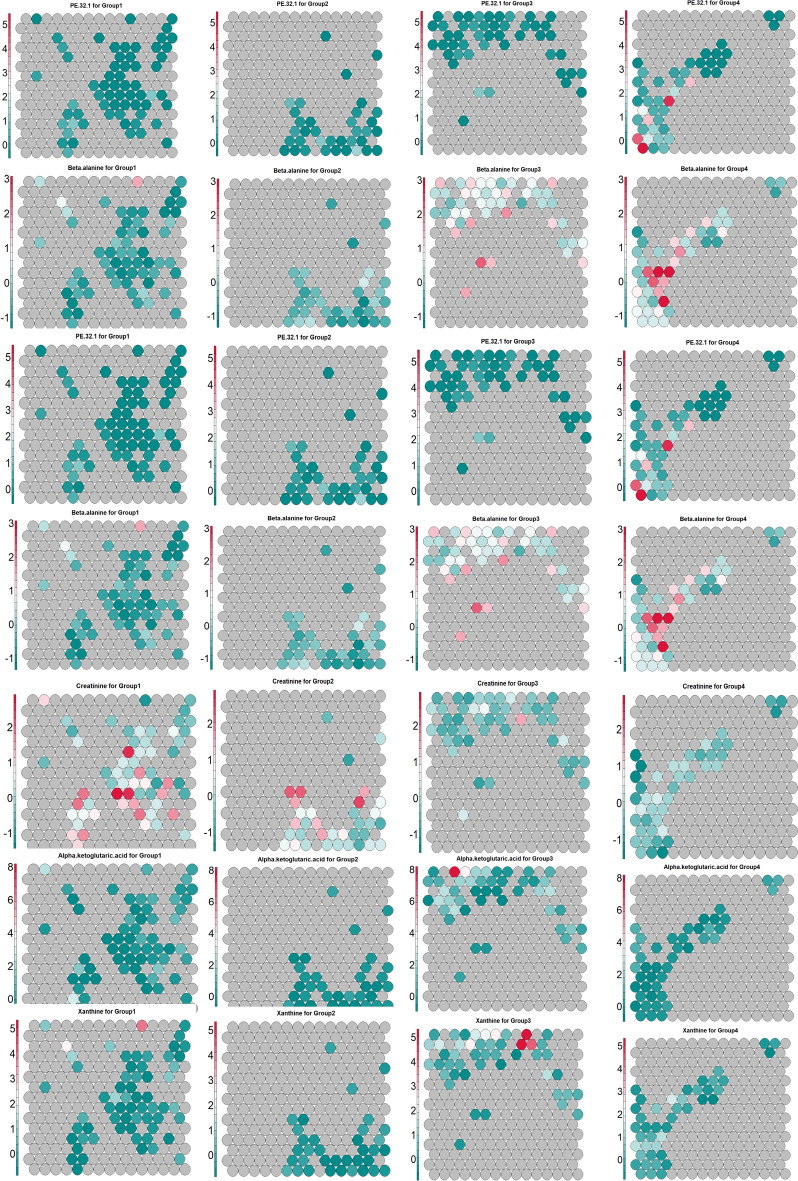
Distribution of metabolite contributions among groups visualized through SOM-based heatmap analysis. Elevated levels of α-ketoglutaric acid, xanthine, maltotriose, and glutamic acid are observed in Group 3. Threonic acid shows a positive contribution in Group 1, while creatine is increased in both Group 1 and Group 2. Malate levels are elevated in Group 3, followed by Group 4. Phosphocholine intensity is highest in Group 4, with slightly lower levels in Group 3. β-Alanine demonstrates a negative contribution in Groups 1 and 2. The color range from red to dark cyan indicates positive contributions, while gray represents neutral or zero contributions, and the cyan range denotes negative contributions.

**Fig. 8:**
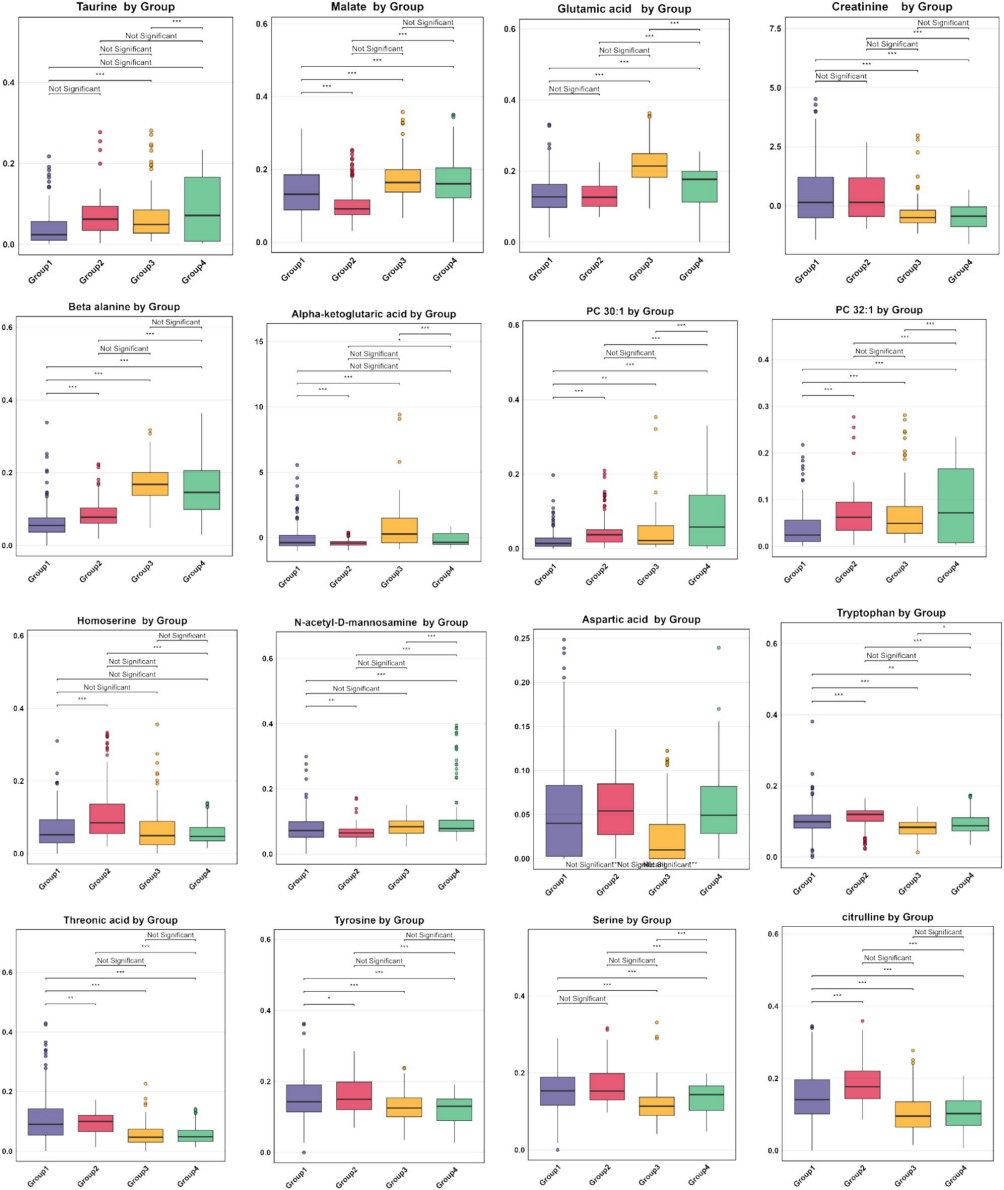
Comparative analysis of scaled metabolite levels across groups with median box plot values and adjusted p-value significance annotations. In Groups 1 and 2, threonine acid, tyrosine, and serine were significantly higher compared to the other groups. Citrulline and homoserine levels were elevated in Group 2. α-Ketoglutaric acid levels increased in Group 3, while β-alanine levels decreased in Groups 1 and 2. Adjusted p-values (Holm–Bonferroni method) were categorized and represented as follows: p < 0.001 as ***, p < 0.01 as **, p < 0.05 as *, and p ≥ 0.05 as ‘Not Significant’.

Figure 7 presents a heatmap illustrating the contributions of key metabolites within the SOM model, subsequently, Figure 8 provides statistical validation of these metabolite profiles across the four groups, using median values and adjusted p-values. In Group 4, elevated levels of mono-unsaturated phosphocholine and phosphoethanolamine are observed, with Group 3 showing slightly lower levels. Conversely, Groups 1 and 2, which are ER or PR positive, display the lowest concentrations of these metabolites. Taurine, glutamine, aspartic acid, and N-acetyl-D-mannosamine exhibit notably higher contributions in the HER2-rich group, with uridine-5-monophosphate also showing increased intensity in this group compared to the others. In the triple-negative group (Group 3), elevated levels of malate, maltotriose, glutamic acid, beta-alanine, alpha-ketoglutaric acid, and xanthine are evident. Meanwhile, Group 2 stands out for its prominent contributions from homoserine, citrulline, and tryptophan. Threonic acid, tyrosine, and serine show higher levels in both Group 1 and Group 2, while 2-aminoadipic acid is uniquely elevated in Group 1.

## Discussion

**Fig. 9:**
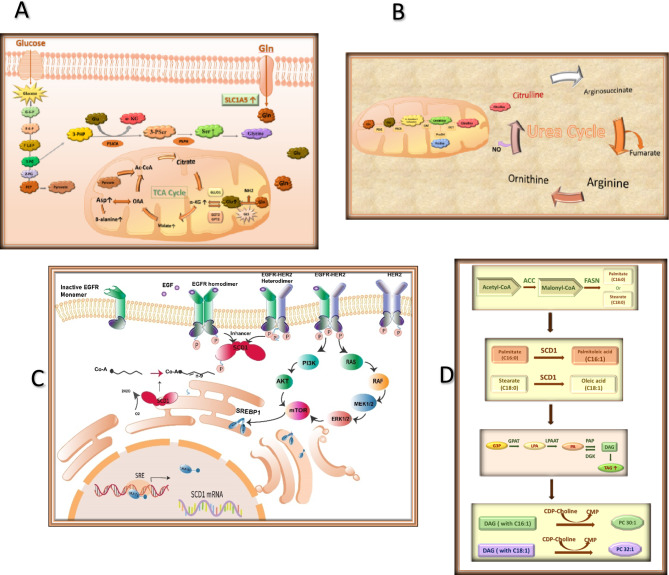
Key metabolite pathways in breast cancer subtypes. (**A**) Serine, glutamic acid, and glutamate levels increase in the Amino Acid Metabolism pathway, contributing to the Warburg effect, activation of the TCA cycle, and elevated levels of α-ketoglutaric acid. (**B**) Citrulline formation in the mitochondria, elevated in Group 2, contributes to the urea cycle and arginine synthesis. (**C**) HER2 and EGFR receptors enhance SCD1 expression by converting saturated phospholipids into monounsaturated phosphocholine and phosphoethanolamine. This process is accompanied by an increased abundance of SREBP1, a transcription factor initially synthesized as pre-SREBP1 in the endoplasmic reticulum (ER). It undergoes proteolytic cleavage by S1P and S2P in the Golgi apparatus, releasing the active nSREBP1 fragment, which translocates to the nucleus and binds to the sterol regulatory element-1 (SRE1) on DNA. This panel is reused from our previously published article in Scientific Reports (Shahnazari et al., 2024) under the Creative Commons Attribution 4.0 International (CC BY 4.0) license (**D**) Formation of Diacylglycerol (DAG), TAG, and monounsaturated phosphocholines (PC 30:1 and PC 32:1). Abbreviations of Key Compounds and Enzymes: SCD1: Stearoyl-CoA desaturase-1; SREBP1: Sterol regulatory element-binding protein 1; EGFR: Epidermal growth factor receptor; DGAT: Diacylglycerol acyltransferase; G3P: Glycerol-3-phosphate; LPA: Lysophosphatidic acid; GPAT: Glycerol-3-phosphate acyltransferase; LPAAT: LPA acyltransferase; DGK: Diacylglycerol kinase; mTOR: Mammalian target of rapamycin; FASN: Fatty acid synthase; SLC1A5: Solute carrier family 1 member 5.

In this study, feature selection on the concatenated data was dynamically applied through an F-ADL model, which identified highly relevant metabolites. The F-ADL model outperformed traditional static feature selection methods, such as SVM-RFE, Random Forest, and XGB-RFE, across multiple evaluation metrics, demonstrating its robust capability to discern key metabolic features. Comparative analysis in Venn’s diagram revealed that the F-ADL model identified more shared metabolites than the static models, suggesting a core set of relevant features consistently detected across different modeling approaches. Interestingly, the F-ADL model also identified fewer unique metabolites than the static methods, indicating a more streamlined feature set focused on the most informative metabolites. The selected features were analyzed using the SOM method, which accurately clustered the data into four distinct multiclass classifications. This clustering revealed clear separation among the class labels; however, Groups 1 and 2 showed some overlap within Group 4. Distinct biomarker patterns and unique metabolite profiles were identified across the four groups using SOM classification, revealing specific metabolic characteristics within each subtype. Group 1, characterized by ER or PR positive and HER2 negative status, displayed a biomarker pattern defined by elevated levels of 2-aminoadipic acid, threonic acid, serine, and tyrosine, with notably low β-alanine levels distinguishing it from the other groups. Group 2 exhibited a metabolite signature that included homoserine and citrulline, while also sharing key markers with Group 1, such as threonic acid, serine, and tyrosine. Distinct biomarker patterns and unique metabolite profiles emerged across the four groups using SOM classification, defining specific metabolic signatures within each subtype. In the TNBC-associated Group 3, elevated levels of α-ketoglutaric acid, glutamate, malate, maltotriose, and β-alanine distinguished its profile. By contrast, the HER2-enriched Group 4 exhibited a unique metabolic composition characterized by high concentrations of N-acetyl-D-mannosamine, glutamine, aspartic acid, and taurine, along with elevated levels of phosphocholines (PC 30:1, PC 32:1, PC 32:2) and phosphatidylethanolamine (PE 32:1). Notably, mono-unsaturated phospholipids in Group 4 showed significant positive contributions, following the TNBC subtype. Conversely, Groups 1 and 2 exhibited negative contributions for the selected metabolites. Enrichment analysis of SOM for each group and heatmaps depicting key metabolite intensities confirmed significant alterations in metabolite profiles across the subtypes. The differential abundance of these metabolites was statistically validated, further supporting the enrichment and heatmap results. The model illustrates the metabolic reprogramming in each group, as shown schematically in Figure 9. Most metabolites identified in the SOM model were consistent with findings in breast cancer, particularly within specific subtypes, and align with previous studies, summarized as follows:

Group 1 exhibited upregulation in amino acid metabolism pathways, indicated by increased levels of serine, tyrosine, and 2-aminoadipic acid (Figure 9A)^19–21^. This pattern suggests an enhancement in amino acid metabolism that may support nucleotide synthesis and maintain redox balance, both crucial for tumor growth and proliferation. In Group 2, elevated levels of homoserine and citrulline imply increased anaplerotic reactions that likely facilitate rapid cell proliferation in HER2-positive environments (Figure 9B)^22–24^**.** Group 3 was distinguished by elevated α-ketoglutarate, glutamate, and malate, signaling an active tricarboxylic acid (TCA) cycle (Figure 9A)^25^. This metabolic profile appears to satisfy the high energy and biosynthetic requirements of cancer cells. Additionally, increased β-alanine and maltotriose levels point to enhanced glycolytic and gluconeogenic activity, further supporting glucose and energy metabolism essential for rapid cell proliferation^26^,Jan^27,28^. Key enzymes such as glutaminase (GLS), which converts glutamine to glutamate to fuel the TCA cycle, and malate dehydrogenase (MDH), which elevates malate levels, play essential roles in sustaining this metabolic state^29^. Elevated levels of α-ketoglutarate and glutamate also underscore a dependence on glutamine, often termed “glutamine addiction,” characteristic of TNBC, enabling continuous energy production and growth by feeding the TCA cycle (Figure 9A).

the prominent phospholipid biosynthesis pathway—marked by high levels of specific PC and PE species, such as PC 30:1, PC 32:1, PC 32:2, and PE 32:1—indicates increased membrane lipid synthesis. This pathway is likely driven by the upregulation of stearoyl-CoA desaturase 1 (SCD1), an enzyme that facilitates the formation of unsaturated phospholipids, enhancing membrane fluidity and stability. This lipid biosynthetic activity supports rapid proliferation and membrane adaptability, key features of HER2-positive cancer cells (Figures 9C and 8D) (10, 11). Moreover, triacylglycerol levels display distinct trends across groups, particularly in aggressive subtypes such as TNBC and HER2-enriched cancers, where TAG accumulation is more pronounced. This may reflect a metabolic shift toward fatty acid storage and TAG droplet formation, potentially acting as a reservoir for energy and metabolic needs during rapid proliferation. In contrast, Group 1 and Group 2 exhibit lower TAG levels, likely due to high TAG consumption to meet the energy demands associated with cell division and growth^30,31^. The formation of mono-unsaturated phosphocholine and triacylglycerol across subtypes is summarized in Figure 9D.

Understanding the metabolomic profiles of each subtype could inform tailored dietary strategies for breast cancer patients. Moreover, developing safe analogs of metabolites like α-ketoglutarate or β-alanine may enhance intraoperative visualization, particularly for TNBC and HER2-enriched cancers. The identified metabolites offer potential biomarkers for diagnosis, targeted therapy, and surgical applications.

The metabolite signature identified in this study, derived from F-ADL and subsequent SOM analysis, is biologically relevant and indicates distinct metabolic processes associated with different breast cancer subtypes. However, further validation using additional datasets is necessary to confirm these findings and establish a more comprehensive metabolic profile across breast cancer subtypes. In addition, using potential biomarkers must consider each cancer subtype’s unique metabolic characteristics and undergo rigorous clinical testing. For instance, in ER/PR-positive, HER2-negative breast cancer, where β-alanine levels are low, supplementation could enhance pH buffering and antioxidant defenses, potentially inhibiting tumor growth adaptations. Conversely, in triple-negative breast cancer (TNBC) and HER2-positive cancers, where β-alanine levels are already elevated, additional supplementation might further activate metabolic pathways that support tumor survival and proliferation.

Despite the promising outcomes of this study, certain limitations warrant consideration. A primary challenge was the lack of publicly accessible datasets containing identical samples used across multiple metabolomics platforms, which provide consistent class labels necessary for data integration. This remains a significant limitation for using external validation. Independent validation using external cohorts is crucial for confirming the robustness and generalizability of the identified metabolic signatures. Nonetheless, this study establishes a synergistic strategy for integrated multimodal metabolomics analysis, facilitating the robust and scalable integration of high-dimensional biological data across various omics layers, thereby providing reliable biomarker discovery and valuable insights into the metabolic underpinnings of disease subtypes.

## Method details

### Study Population and Histopathological Examination.

The datasets analyzed in this study were derived from breast cancer tissue samples originally collected through the METAcancer FP7 project^32,33^. The METAcancer initiative aimed to determine whether alterations in metabolite levels could contribute to the molecular classification of breast cancer and support the identification of novel prognostic and predictive biomarkers. To achieve broad metabolomic coverage, the project employed three distinct profiling technologies: High-Resolution Magic Angle Spinning Proton Nuclear Magnetic Resonance (^1^H NMR), Gas Chromatography–Time-of-Flight Mass Spectrometry (GC-TOFMS), and Liquid Chromatography–Mass Spectrometry (LC-MS). The METAcancer project produced the datasets but only analysed results using a single metabolomics platform dataset at a time. In the present study, we integrate various cross-platform metabolomics data (data fusion) before analysis and investigate subtype-specific metabolic signatures. Originally, the breast cancer samples were categorized into four groups based on their HER2, estrogen receptor (ER), and progesterone receptor (PR) status as follows:

- **Group 1**: ER and/or PR positive, HER2 negative
- **Group 2**: ER, PR, and HER2 positive
- **Group 3**: Triple-negative (ER, PR, and HER2 negative)
- **Group 4**: HER2-enriched (HER2 positive, ER and PR negative)

There are limitations with such classification, as it does not fully correspond to conventional molecular subtypes, with key markers such as Ki-67 and P53 not included in the provided metadata^34^. Table 2 summarizes the number of samples and metabolite features across each analytical platform (detailed metadata shared via FigShare). To ensure comparability, the original datasets were curated by retaining only samples with confirmed hormone receptor and their HER2 status that were present across all three platforms. The resulting subtype distribution revealed an imbalance across the datasets that was addressed using SMOTE (See below).

**Table 2:** Table 2: Summary of sample counts and metabolite coverage across the three analytical platforms.

Metric	LC–MS	GC–MS	NMR
Initial Sample Count	426	300	355
Subtype-Matched Sample Count	253	253	253
Total Metabolites (Known + Unknown)	426	220	793*
Annotated Metabolites	210	161	180
Group Distribution			
Group1	175	175	175
Group2	31	31	31
Group3	19	19	19
Group4	18	18	18

The "Initial Sample Count" represents the raw samples before subtype filtering, while the "Subtype-Matched Sample Count" reflects the number of samples matched across all three platforms. “Total Metabolites” includes known and unknown metabolites, and “Annotated Metabolites” shows identified metabolites. The distribution of samples across four groups (Group 1 to Group 4) is also provided. NMR includes 793 spectral features, which comprise both annotated and unannotated signals. These features represent known metabolites, unknown compounds, and spectral fragments such as baseline regions, overlapping peaks, and signals across various chemical shifts (ppm). Due to the inherent complexity of NMR spectra and peak overlaps, a portion of these features remains unassigned.

### Data curation, data preprocessing, data oversampling and concatenation

Metabolite profiling in this study employed three analytical platforms—NMR, GC-MS, and LC-MS (positive mode)—to capture complementary metabolic features across four breast cancer subtypes. The datasets were curated to retain only samples measured on all three platforms, resulting in 253 matched biological samples with platform-specific feature counts (NMR: 180, GC-MS: 161, LC-MS-positive: 183). After missing-value imputation, each platform-specific dataset was independently min–max scaled to [0, 1] and row-wise L2-normalized to unit length to ensure cross-platform compatibility (see 2-2). To address class imbalance across the four subtypes, we then applied the Synthetic Minority Over-sampling Technique (SMOTE; scikit-learn implementation in Python –^35^), expanding each dataset from 253 to 740 samples (185 per subgroup) while preserving biological consistency. Since all platforms shared identical sample identifiers, horizontal concatenation of the 740-sample datasets was then feasible, yielding a unified matrix with integrated features from all three platforms. Finally, the concatenated dataset was standardized via z-score transformation (zero mean, unit variance) based on statistics computed from the training set, preparing the data for downstream machine-learning analyses.

### Feature selection

Static feature selection contains, SVM-RFE, XBG-RFE, and RF.

### SVM-RFE, XGB-RFE and RF

SVM-RFE is a feature selection method that iteratively removes the least significant features based on their contribution to an SVM model, thereby optimizing predictive accuracy^36^. This study used a linear kernel SVM model (C=1) and repeated stratified cross-validation using k=5 fold. XGB-RFE is a similar SVM-RFE feature selection technique that combines the predictive power of XGBoost with recursive elimination. It ranks features by their importance scores from an XGBoost model, iteratively removing the least important features and refitting the model until the optimal subset of features is identified^37^. . In this study, hyperparameter tuning was first performed using GridSearchCV with Stratified 5-fold cross-validation to optimize XGBoost parameters, including estimators, depth, learning rate, subsampling, and regularization terms, to enhance model performance. Following tuning, the XGB-RFE method was applied to select key features by iteratively removing those with the lowest importance. This combined approach refined the model’s feature set and maximized accuracy, interpretability, and robustness across multiclass classifications. Random Forest feature selection leverages the inherent ability of the Random Forest algorithm to rank features by importance based on their contribution to prediction accuracy. By constructing multiple decision trees and aggregating their results, Random Forest assigns an importance score to each feature, reflecting its role in reducing classification error. This method aids in identifying the most relevant predictors, allowing researchers to filter out less informative features and improve model performance and interpretability, especially in high-dimensional datasets^38^.

Hyperparameter tuning for the RF was conducted using GridSearchCV with 5-fold cross-validation. The tuning focused on optimizing key parameters: the number of estimators, tree depth, maximum features per split, minimum samples required to split a node, and minimum samples required at each leaf. The goal was to identify the combination that maximizes the macro F1 score, ensuring robust model performance across classes. The cross-validation approach helped estimate performance stability, and the best configuration was used for feature selection and final model evaluation.

### Feedforward attention-based deep learning (F-ADL)

The feedforward attention-based deep learning model is implemented using TensorFlow Keras libraries, focusing on dynamically selecting relevant features through an attention mechanism. The model architecture includes attention layers alongside dense layers optimized for feature selection. To prevent overfitting, L2 regularization, batch normalization, and dropout layers are incorporated into the dense layers. Hyperparameter tuning is conducted using RandomizedSearchCV from Keras-tuner, with values explored for dropout rates (0.6, 0.7, 0.8), L2 regularization coefficients (0.05, 0.1, 0.2), number of epochs (50, 100), and batch sizes (32, 64). The best configuration is selected based on the model’s performance. Cross-validation with Repeated Stratified K-Fold is used for robust model evaluation, and early stopping is applied to monitor validation loss and prevent overfitting. The model is trained and evaluated on key metrics such as accuracy, F1 scores, and AUC^39^.

### Self-organizing maps

In the supervised SOM, the data was split into training (70%) and test (30%) sets. The SOM model was trained using the *supersom* function from the *kohonen* package in R with a 15x15 hexagonal grid^40^. The training parameters included 1,000 iterations, an initial learning rate (alpha) decreasing from 0.1 to 0.01, and a neighborhood radius decreasing from 4 to 0.5, with the "sum of squares" distance function. Predictions for the test data were assigned based on the majority class labeling of SOM units, derived from training data mappings.

### Model validation

The performance of the multiclass label classification was evaluated using 5-fold repeated cross-validation (repeated-CV) to ensure robust model assessment and facilitate meaningful comparisons between feature selection models. Given the data’s imbalanced and multiclass characteristics, we selected specific metrics to provide a comprehensive assessment. The evaluation metrics included Accuracy, Balanced Accuracy, Macro F1, Micro F1, Weighted F1 Score, Hamming Loss, AUC, and MCC. Macro F1 and Micro F1 scores were chosen to capture balanced performance across classes: Macro F1 treats each class equally, which is essential for understanding performance in underrepresented classes, while Micro F1 aggregates performance across all samples, placing greater emphasis on more frequently occurring classes. Hamming Loss was used to quantify the proportion of incorrect predictions, making it particularly valuable for identifying errors across multilabel classifications. The MCC metric was included as it considers true and false positives and negatives, providing a balanced assessment that is especially valuable for evaluating performance in imbalanced multiclass settings, where equal consideration of all classes is critical.

### Statistical analysis

Median box plots were used to compare individual metabolite levels among groups to distinguish selected metabolites across the classification groups. Adjusted p-values (Holm–Bonferroni method) were categorized and represented as follows: p < 0.001 as ***, p < 0.01 as **, p < 0.05 as *, and p ≥ 0.05 as ‘Not Significant’.

## Data Availability

Data and Code Availability The LC–MS. GC–MS and NMR data used during this study are deposited and publicly available at https://doi.org/10.6084/m9.figshare.28418267 under the data folder. To promote transparency and reproducibility, all research data and associated code are openly accessible on the CodeOcean platform. A Compute Capsule has been provisionally assigned the URL “https://doi.org/10.24433/CO.7561930.v1.”
